# Fabry-Perot cavity resonance enabling highly polarization-sensitive double-layer gold grating

**DOI:** 10.1038/s41598-018-32158-y

**Published:** 2018-10-03

**Authors:** Jehwan Hwang, Boram Oh, Yeongho Kim, Sinhara Silva, Jun Oh Kim, David A. Czaplewski, Jong Eun Ryu, Eun Kyu Kim, Augustine Urbas, Jiangfeng Zhou, Zahyun Ku, Sang Jun Lee

**Affiliations:** 1 Division of Industrial Metrology, Korea Research Institute of Standards and Science, Daejeon, 34113 Korea; 2 Department of Physics, Hanyang University, Seoul, 04763 Korea; 3 Department of Mechanical Engineering, Indiana University–Purdue University Indianapolis, Indianapolis, IN 46202 USA; 4 Department of Physics, University of South Florida, Tampa, FL 33620 USA; 5 Center for Nanoscale Materials, Argonne National Laboratory, 9700 S. Cass Ave., Argonne, IL 60439 USA; 6 Department of Mechanical and Aerospace Engineering, North Carolina State University, Raleigh, NC 27695 USA; 7 Materials and Manufacturing directorate, Air Force Research Laboratory, WPAFB, OH 45433 USA

## Abstract

We present experimental and theoretical investigations on the polarization properties of a single- and a double-layer gold (Au) grating, serving as a wire grid polarizer. Two layers of Au gratings form a cavity that effectively modulates the transmission and reflection of linearly polarized light. Theoretical calculations based on a transfer matrix method reveals that the double-layer Au grating structure creates an optical cavity exhibiting Fabry-Perot (FP) resonance modes. As compared to a single-layer grating, the FP cavity resonance modes of the double-layer grating significantly enhance the transmission of the transverse magnetic (TM) mode, while suppressing the transmission of the transverse electric (TE) mode. As a result, the extinction ratio of TM to TE transmission for the double-layer grating structure is improved by a factor of approximately 8 in the mid-wave infrared region of 3.4–6 μm. Furthermore, excellent infrared imagery is obtained with over a 600% increase in the ratio of the TM-output voltage (*V*_*θ* = 0°_) to TE-output voltage (*V*_*θ* = 90°_). This double-layer Au grating structure has great potential for use in polarimetric imaging applications due to its superior ability to resolve linear polarization signatures.

## Introduction

Infrared (IR) imaging systems have been widely used in many areas, especially remote sensing, surveillance, and medical diagnosis^1^. Over the past years, there have been considerable efforts in the development of IR sensors for advanced imaging systems such as multicolor functionality, polarization imaging and high frame rate operation. Conventional IR imaging systems typically use reflected light and emitted thermal radiation from objects in the short-wave (1.4–3 μm), the mid- (3–5 μm) and the long-wave (8–14 μm) IR regions to generate thermal intensity images. Recently, the polarimetric imaging system has attracted much attention as one of the fourth generation IR imaging systems because of its capability to collect polarization signatures from a scene of interest^2,3^. Compared to physical quantities such as intensity and wavelength of light, polarization, which is described as the orientation of the transverse electric field, has not been utilized in IR imaging systems. The use of polarization enables the detection and identification of targets from cluttered backgrounds with no thermal contrast by discriminating the polarization states of emitted and/or reflected light, which are determined by their geometry and surface roughness^3,4^. In addition, surface orientation and texture of objects can be characterized by measuring the degree of polarization (DoP) of light, given by the ratio of the polarized light intensity to the total light intensity^2,5^. The DoP varies between 0 (completely unpolarized light) and 1 (completely polarized light). Moreover, the polarization-based imaging technique is very useful in the classification of materials such as metals and dielectrics^6^. This is because the DoP of the specularly reflected light from a dielectric surface is much larger than that from a metal surface. Usually, polarimetric imaging is implemented by adjusting a conventional linear polarizer and a quarter-wave plate in front of an imager to obtain the polarimetric contrast between objects and backgrounds. However, this imaging system is bulky, clumsy, and incapable of acquiring polarimetric images in real time.

Recent advances in micro/nanofabrication techniques enable on-chip integration of micro-/nanoscale materials and devices^7,8^. For instance, subwavelength one-dimensional metal line arrays (a single-layer 1D grating acting as a wire grid polarizer) capable of being integrated on a chip offers a high extinction ratio, which is defined as the ratio of the transmitted intensity of TM-polarized light to that of TE-polarized light due to high polarization sensitivity in a broad range of wavelength and angles of incidence^8^. High transmission efficiency can also be obtained by strong coupling of the evanescent fields on both grating surfaces under appropriate conditions and by waveguide resonances inside the slits of the grating^9^. A pixelated microgrid polarizer array with various types of single-layer elements such as metallic grating, liquid-crystal guest-host, and elliptical Si nanowire is integrated into complementary metal-oxide-semiconductor (CMOS) image sensors and Si photodetectors for polarimetric imaging^10–16^. The pixelated imaging system uses a superpixel consisting of a 2 × 2 array of four adjacent pixels with 0°, 45°, 90°, and 135° orientated 1D gratings in order to collect the intensity information of polarized light, and thereby the three Stokes parameters, namely *S*_0_, *S*_1_, and *S*_2_ for linear polarization, can be estimated. The performance of the microgrid polarizer is further enhanced by employing two or more layers of subwavelength 1D gratings. It is known that a strong coupling between the evanescent fields on the surfaces of layered metals results in a high TM transmission^17^. Additionally, TM transmission and the incidence angle that gives the maximum TM transmission can be controlled by adjusting the lateral shift between the layered metal layers and the grating pitch, respectively^17–19^.

In this work, we investigate the transmission and polarization properties of single-/double-layer subwavelength Au gratings. It is demonstrated experimentally and theoretically that the double-layer Au grating shows superior transmission properties (i.e., high TM transmission and low TE transmission) as compared to the single-layer Au grating. A multiple-layer model based on the transfer matrix method reveals that both the TM transmission and the extinction ratio of the double-layer Au grating can be improved by selecting an appropriate thickness of a dielectric embedded between two layers of the Au grating, leading to a Fabry-Perot cavity resonance. In addition, radiometric measurement with our fabricated 320 × 256 type-II InAs/GaSb superlattice IR focal plane array (FPA) sensor validates that the double-layer Au grating produces IR images with much improved extinction ratio, evaluated from the output pixel values for TM- and TE-polarizations. Our developed multiple-layer model is very widely applicable to the design of an upper metal grating-dielectric-lower metal grating system capable of exciting Fabry-Perot cavity resonance by carefully controlling the propagating phase factor of dielectric spacer.

## Fabrication of double-layer Au grating

**Figure 1:**
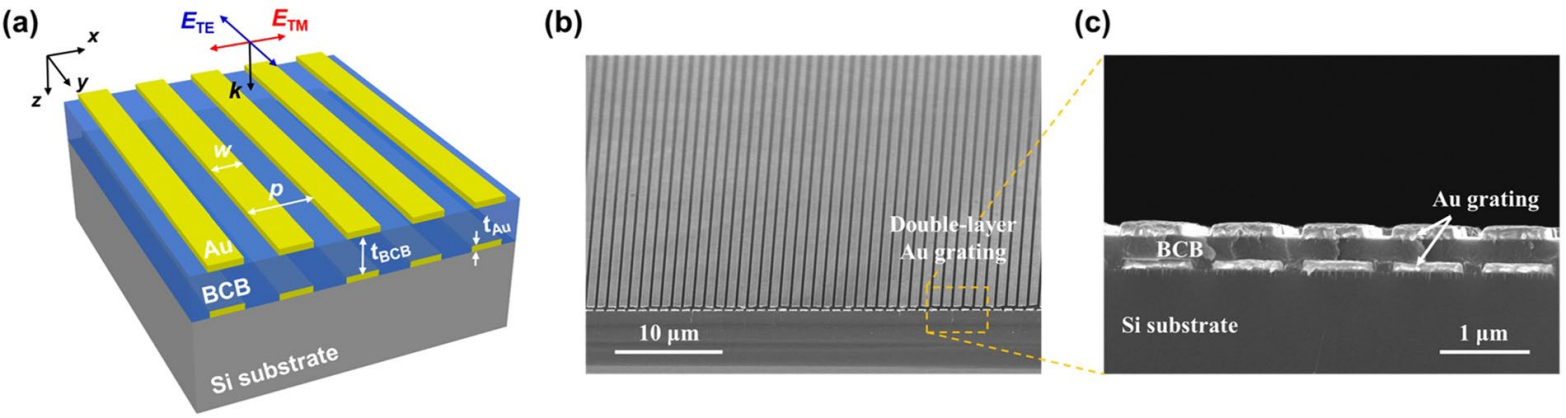
(**a**) Schematic illustration of the double-layer Au grating composed of the dielectric spacer situated between identical Au gratings. Here, *p* is the Au grating periodicity (1 μm), *w* is the Au grating width (0.7 μm), *t*_*Au*_ is the metal thickness (0.1 μm), and *t*_*BCB*_ is the BCB thickness (0.25 μm) between the gratings. (**b**) Tilted-view and (**c**) cross-sectional-view scanning electron microscope (SEM) images of the double-layer Au grating.

Double-layer gold (Au) grating structure was fabricated on a Si substrate using a UV-nanoimprint lithography (UV-NIL) process. The geometry and dimensions of the double-layer Au grating structure are illustrated in Fig. 1a: periodicity (*p*) = 1.0 μm; width (*w*) = 0.7 μm; and Au thickness (*t*_*Au*_) = 0.1 μm. A 300 nm-thick polymethylmethacrylate (PMMA) resist was spin-coated on a Si substrate and soft-baked at 180°C on a hotplate for 90 sec. A UV-curable NIL resist (UVP, EZimprinting) was spun on top of the PMMA for the NIL process. A polydimethylsiloxane (PDMS) mold with the grating pattern was imprinted onto the UV resist at a pressure of 10 PSI for 2 minutes using a commercial nanoimprinter (PL-400, EZimprinting), followed by UV radiation curing. After demolding, the residual UV resist and PMMA were anisotropically etched by CHF_3_/O_2_ and O_2_ plasma in a reactive-ion-etch (RIE), respectively. An Au layer was deposited by electron beam evaporation of Cr (5 nm)/Au (100 nm) and lift-off. The same NIL process was repeated to fabricate the upper Au grating, which was vertically aligned with the lower one. The detailed fabrication can be found in Figs S1, S2 (Supplementary Information). The upper and lower Au gratings were separated by a dielectric spacer of 0.25 μm-thick benzocyclobutene (BCB). BCB has the advantages of a frequency-independent refractive index (*n* ≈ 1.54) with a low loss (*k* ≈ 10^−4^) in the mid-wave infrared region (MWIR, 3–5 μm), a good adhesion to metal and semiconductor, and a low curing temperature (180–250 °C). Figure 1c shows the cross-sectional SEM image of the fabricated double-layer Au grating. Notably, the spin-coated BCB on the lower Au grating has an excellent flatness, which enables high alignment accuracy and good pattern fidelity for the fabrication of the upper Au grating.

## FTIR-transmission contrast of the fabricated single- and double-layer Au gratings

**Figure 2:**
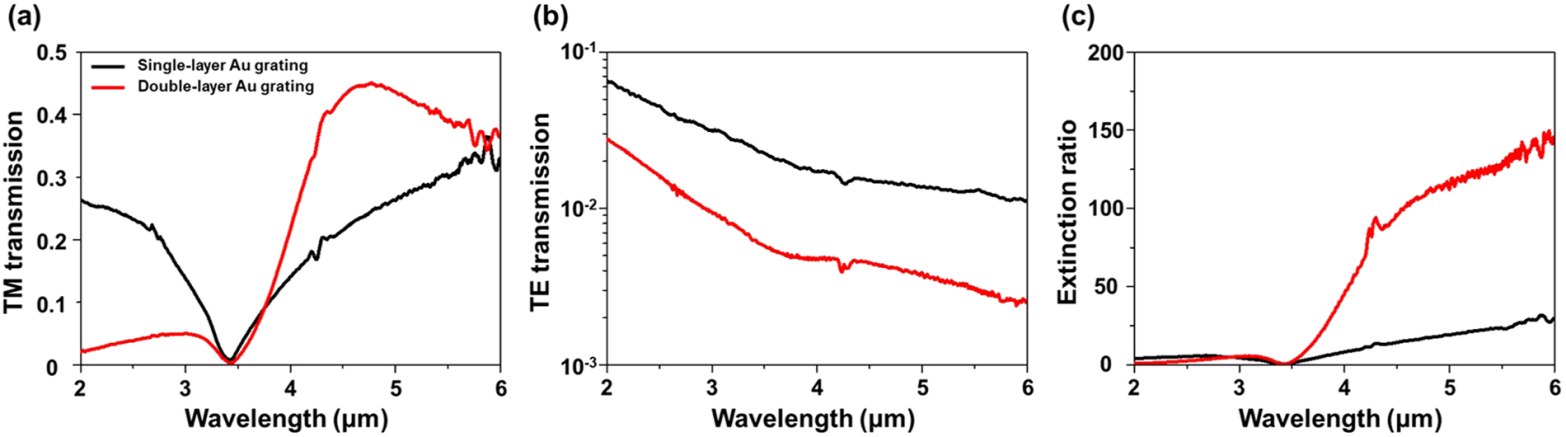
FTIR measured transmission spectra of (**a**) TM and (**b**) TE-polarized incident light and (**c**) extinction ratio of the single- and double-layer Au grating structures (*p* = 1.0 μm, *w* = 0.7 μm, *t*_*Au*_ = 0.1 μm, *t*_*BCB*_ = 0.25 μm).

The transmission spectra of both single- and double-layer Au gratings for normally incident TE and TM polarized lights were measured in the wavelength range of 2–6 μm by an Agilent Technologies Cary 670 Fourier transform infrared (FTIR) spectrometer. As shown in Fig. 1a, the TE (TM) polarization is defined as a polarization state, where the electric field orientation is parallel (perpendicular) to the Au grating. The FTIR beam was linearly polarized along either the TE or the TM direction by a commercial wire grid polarizer and the FTIR-measured transmission spectra were recorded with a resolution of 2 cm^−1^ using a liquid nitrogen-cooled mercury-cadmium-telluride detector. Figure 2a,b show the measured transmission spectra for the TM and TE modes of single- and double-layer Au grating structures. Distinctive transmission dips in the TM transmission spectra for both single- and double-layer Au grating structures are observed at ~3.4 μm, which are caused by the first-order surface plasmon polariton (SPP) resonances at the interface between the lower Au grating and the Si substrate. The TM transmission of a single-layer Au grating structure increases monotonously in the MWIR region of 3.4–6 μm. Meanwhile, TM transmission of a double-layer Au grating structure in the MWIR region increases quickly, and then decreases relatively slowly after reaching a peak transmission at ~4.5 μm. The experimental TM transmission spectrum of the double-layer Au grating will be compared with the theoretical calculations in the following discussion. In the region of 3.4–6 μm, the double-layer Au gratings structure exhibits a higher TM transmission over the wavelength region of interest compared to the single-layer Au grating structure. In Fig. 2b, TE transmission of the double-layer Au grating structure is more than a half order of magnitude lower than that of the single-layer Au grating structure. This is due to the sequential attenuation of the electric field (*E*_*y*_) of the incident radiation through the upper and lower Au gratings, caused by a reflection from the Au grating surfaces and a restricted propagation of the TE modes through the subwavelength periodic 1D arrays^7^. The extinction ratio, used as a performance indicator of the gratings, is calculated from the ratio of the TM to the TE transmission. As indicated in Fig. 2c, the double-layer Au grating structure offers nearly eight times higher extinction ratio than the single-layer Au grating structure in the wavelength range of 2–6 μm. The dips of the extinction ratio spectra at ~3.4 μm due to the SPP resonance, causing a degradation of the extinction ratio, can be shifted to shorter wavelength by reducing the grating period and/or increasing the index of refraction of the spacer (*n* < *n*_*substrate*_ is required)^20^.

## Analysis of double-layer Au grating by a multiple-layer effective medium model

**Figure 3:**
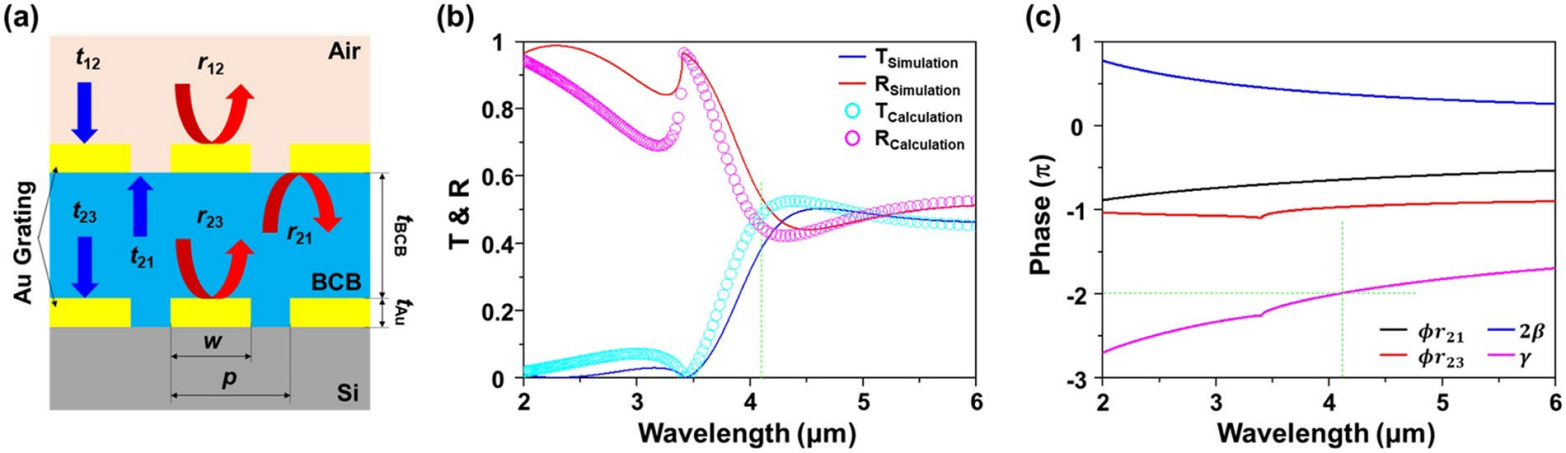
(**a**) Schematic diagram of the transmission and the reflection coefficients of the double-layer Au grating. (**b**) Transmission and reflection spectra obtained by a full-wave simulation (based on FIT) of the actual double-layer Au grating structure and the analytical calculation (using the multiple-layer model). (**c**) Phase terms, $\varphi ({r}_{21})$, $\varphi ({r}_{23})$, 2*β*, *γ* in the FP cavity resonance condition.

A multiple-layer effective medium model was employed to better understand the underlying mechanism of wave propagation inside the double-layer Au grating structure (*p* = 1.0 μm, *w* = 0.7 μm, *t*_*Au*_ = 0.1 μm, and *t*_*BCB*_ = 0.25 μm) as displayed in Fig. 3a. In this model, the gratings are considered as continuous surfaces that reflect the light speculary because the wavelength of light in the BCB layer is much larger than the period of grating *p* in the region of 3.4–6 μm. The overall reflection and transmission coefficients of the double-layer grating depend on the transmission and reflection of each grating layer and can be obtained by a transfer matrix method^21,22^. Using this method, the overall transfer matrix of the entire double-layer grating is calculated by the multiplication of the transfer matrix of each layer

$$M={M}_{upper}\cdot {M}_{BCB}\cdot {M}_{lower}$$

where *M*_*BCB*_, *M*_*upper*_ and *M*_*lower*_ are the transfer matrices for the BCB, the upper and lower grating layers, respectively. The overall reflection and transmission coefficients *r*, *t* as expressed below in equation (1), can be obtained through the correlation between the total transfer matrix, *M*, and *S*-parameters^21,22^,

1 $$r=\frac{{r}_{12}+\alpha {r}_{23}{e}^{-2i\beta }}{1-{r}_{21}{r}_{23}{e}^{-2i\beta }},\,t=\frac{{t}_{12}{t}_{23}{e}^{-i\beta }}{1-{r}_{21}{r}_{23}{e}^{-2i\beta }}$$

where *r*_12_ and *r*_21_ are the reflection coefficients at the front and the back sides of the upper Au grating, respectively. *r*_23_ is the reflection coefficient from the lower Au grating and *β* is the propagating phase factor of $\beta ={n}_{BCB}\cdot k\cdot {t}_{BCB}$ in the BCB layer, where *n*_*BCB*_, *k*, and *t*_*BCB*_ are the refractive index of BCB, the wave vector in free space, and the BCB thickness, respectively. $\alpha \,(\,\approx \,1)$ is given by ${t}_{21}{t}_{12}-{r}_{21}{r}_{12}$, where *t*_12_ and *t*_21_ are the forward and the backward transmission coefficients through the upper Au grating, respectively. Figure 3b shows the reflection *R* and transmission *T* spectra obtained by using equation (1) based on the multiple-layer model and by numerical simulation of the entire double-layer grating. In the multiple-layer model calculation, the transmission and the reflection coefficients for each grating layer are also obtained through numerical simulations of the corresponding individual layers (air/upper Au grating-BCB and BCB-lower Au grating/Si) with more details elaborated in our previous works^23–25^. Our numerical simulations were performed by CST Microwave Studio^26^, which uses a finite integration technique (FIT). In the simulations, the complex dielectric function of Au is assumed to follow the Drude model with a plasma frequency of *ω*_*p*_ = 1.38 × 10^16^ Hz and a collision frequency of *ω*_*c*_ = 5.71 × 10^13^ Hz.

Theoretically calculated spectral shapes of TM-polarized light transmission through the sample (reconstructed transmission by the multiple-layer model and FIT-simulation) show similar trends as the measured one in Fig. 2a, with a transmission dip at ~3.4 μm and a transmission peak at ~4.5 μm. Note that the amplitude discrepancy between the experimental and the theoretical observations for the double-layer Au grating is possibly due to imperfections in the fabrication process. Furthermore, the calculated reflections and transmissions from the multiple-layer model (symbols) are in good agreement with ones by FIT-simulation (solid lines) in the MWIR region (Fig. 3b). A clear deviation of the calculated results from the FIT-simulation results is found in the wavelength region of 2–3.4 μm because of the violation of the effective medium assumption, where the first-order diffraction occurs below 3.4 μm.

The double-layer Au grating structure behaves as a Fabry-Perot (FP) cavity where multiple reflections occur at two interfaces: the air/upper Au grating–BCB and the BCB–lower Au grating/Si. Note that the BCB thickness (*t*_*BCB*_ = 0.25 μm) between the upper and the lower Au gratings was chosen to satisfy the FP cavity condition (equation 2) for increasing the TM transmission intensity, and thereby improving the extinction ratio of the double-layer Au grating structure as compared with the single-layer Au grating structure.

2 $$\gamma =\varphi ({r}_{21})+\varphi ({r}_{23})-2\beta =2m\pi ,\,|m|=0,\,1,\,2,\ldots $$

Constructive interference inside the spacer (between the upper and the lower Au gratings) occurs when the round-trip propagation phase (*γ*) is equal to an integer multiple of 2*π*. As shown in Fig. 3c, the FP resonance condition of *γ* = −2*π* is satisfied at ~4.2 μm, where the transmission reaches the peak value. The enhancement of transmission is due to the maximum reduction of the reflection by destructive interference at the top surface of the upper grating layer when the FP resonance condition is satisfied. *γ* is predominantly governed by the propagating phase factor *β*, and thus the BCB thickness has to be chosen carefully to improve the TM transmission assisted by the FP cavity resonance. Another thing to note is that there is a slight difference (${\rm{\Delta }}\lambda \,\approx$ 0.3 μm) in the wavelength of the peak transmission for simulation and calculation *γ* = −2π), which is probably attributed to the coupling between SPP and FP resonance modes^27^.

**Figure 4:**
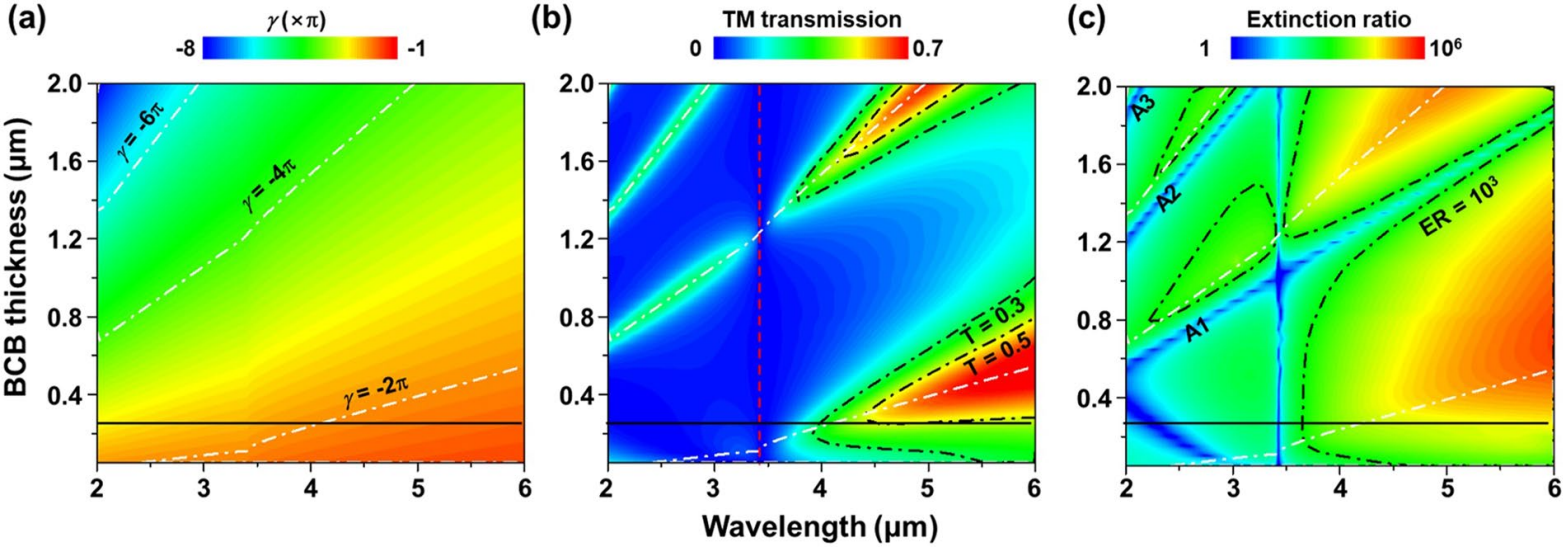
(**a**) Colormap of the round-trip propagation phase, *γ*, as a function of wavelength, *λ*, and BCB thickness, *t*_*BCB*_, for TM-polarized incident light. The white dash-dot lines indicate where the FP cavity resonance condition of $\gamma =-\,2\pi ,\,-\,4\pi ,\,-\,6\pi$ is satisfied. Colormaps of (**b**) TM transmission (red dash line represents the first-order SPP resonance at the interface of the lower Au grating/Si) and (**c**) extinction ratio obtained by CST-simulation of the entire double-layer Au grating structure (blue regions of A1, A2, A3 have low extinction ratio due to the transmission peaks for TE-polarized light). The black-horizontal lines in (**a**–**c**) denote the double-layer Au grating with *t*_*BCB*_ = 0.25 μm.

Figure 4 shows the colormaps obtained by the multiple-layer model for the round-trip propagation phase and the simulation of actual double-layer Au grating structures for TM transmission and extinction ratio as a function of wavelength (2 μm ≤ *λ* ≤ 6 μm) and BCB thickness (0.05 μm ≤ *t*_*BCB*_ ≤ 2 μm). In Fig. 4a, it can be clearly seen that the number of round-trips, $|\gamma |$, is decreased with a fixed spacer thickness (*t*_*BCB*_) as the wavelength increases. As aforementioned,*γ* is highly dependent on *β* (i.e., the refractive index *n*_*BCB*_ and the thickness *t*_*BCB*_ of the dielectric spacer) since the phase terms of *ϕ*(*r*_21_) and *ϕ*(*r*_23_) tend to keep almost unchanged. Accordingly, *n*_*BCB*_ and *t*_*BCB*_ should be considered as important determinants when designing double-layer grating structures. The white dash-dot lines in Fig. 4 indicate where the FP cavity resonance condition for TM-polarized incident light is satisfied. In the wavelength range of 2–6 μm, the spectral position of the TM-transmission peaks for the double-layer Au grating structure is mostly coincident with the position of the FP resonance modes as shown in Fig. 4b. The lowest order of the FP cavity resonance condition (i.e., *γ* = −2π at *t*_*BCB*_ = 0.25 μm) is the most suitable to realize the broadband linear polarizer, i.e., for the spectral response that covers the wavelength range from 3 μm to 5 μm (MWIR region). Hence, this double-layer grating structure can be used as a broadband wire grid polarizer with high transmission efficiency and high extinction ratio. The red dash line indicates the (1, 0) SPP mode at the interface of the lower Au grating and Si substrate, whose resonance wavelength is independent of *t*_*BCB*_. The regions where the extinction ratio is low (blue) are clearly observed in Fig. 4c, marked as A1, A2, and A3. The degradation of the extinction ratio is also associated with the FP resonance occurring between the two layers of Au grating under TE-polarized incident light (not shown here). For TE-polarized incidence, the upper and the lower gratings act as a mirror with a high reflectivity (i.e., $\varphi ({r}_{21})$ and $({r}_{23})\approx \pi$) and thus equation (2) can be simplified to $\beta \approx \pi (1-m)$, where *m* = 0 and negative integers. So the BCB thickness can be derived as ${t}_{BCB}\approx \lambda (1-m)/2{n}_{BCB}$ and *t*_*BCB*_(*λ*) with *m* = 0, −1, −2 is well matched with A1, A2, and A3, respectively.

## MWIR images for the linearly-polarized test target

**Figure 5:**
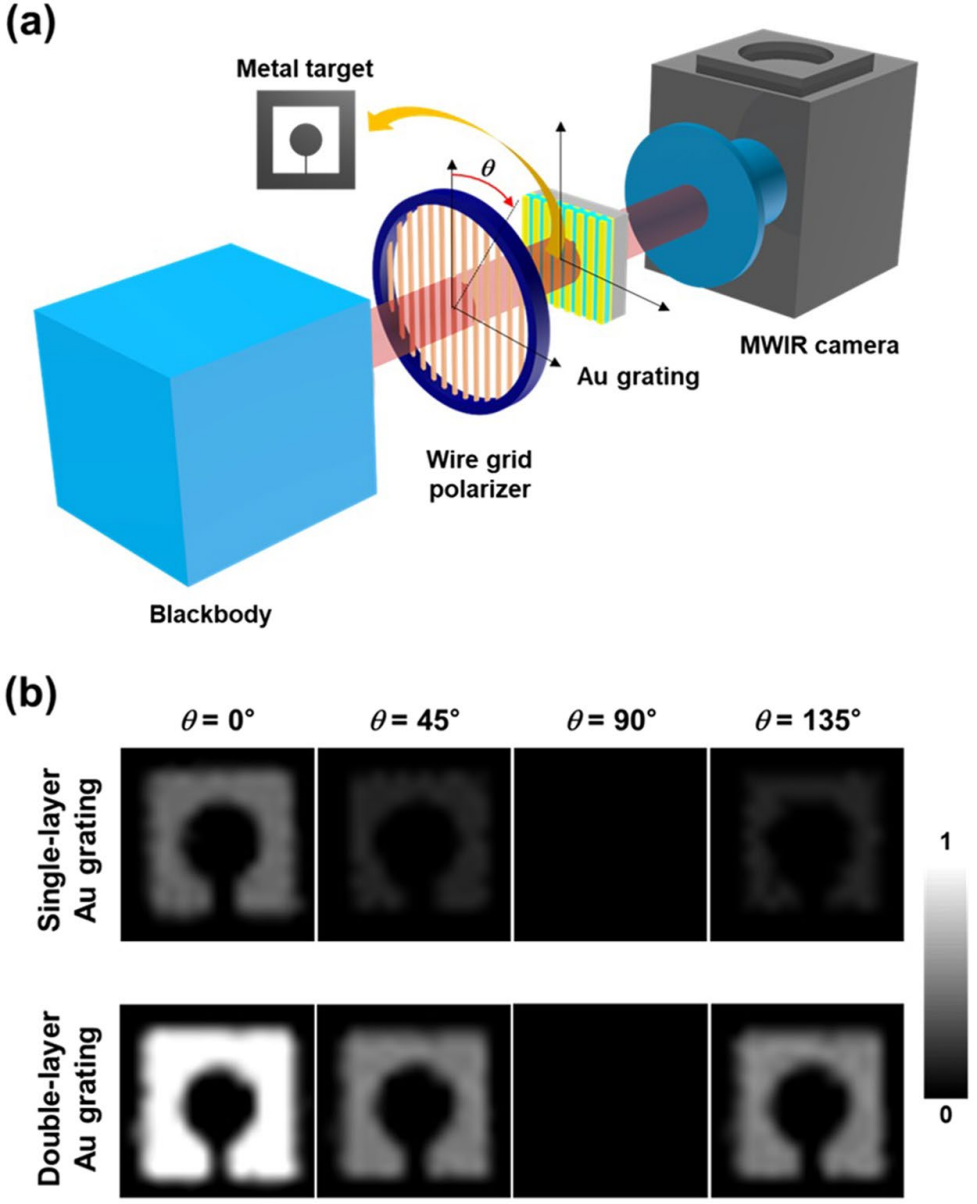
(**a**) The diagram represents the experimental set-up to characterize the linear polarization performance of the single- and the double-layer Au grating structures using an artificially linearly-polarized test target (a blackbody, a commercial wire grid polarizer, and a metal target) and type-II superlattice based MWIR camera. *θ* is the mutual angle of the two polarization axes of the wire grid polarizer and the fabricated Au grating. (**b**) MWIR images captured using the single- and double-layer Au grating samples for test target at $\theta ={0}^{\circ },\,{45}^{\circ },\,{90}^{\circ },\,{135}^{\circ }$.

An IR camera system was used to experimentally compare the linear polarization performance of the single- and the double-layer Au grating structures^28^. The experimental set-up consists of an artificial target with linearly polarized components (a blackbody, a commercial wire grid polarizer, a metal target), the fabricated single- and the double-layer Au grating samples, and a 320 × 256 MWIR type-II InAs/GaSb superlattice based imager. The IR radiation emitted by a blackbody at 300°C is linearly polarized upon passing through the wire grid polarizer, which is followed by transmitting through the metal target and the fabricated sample, sequentially. Finally, it is incident on the detector array of the MWIR camera. In order to capture the IR images with a variety of linear polarization states through the metal target, we define the mutual angle *θ* between two polarization axes of the fabricated Au grating and the wire grid polarizer. The mutual angle *θ* is varied from 0° to 360° with a step of 15°. Figure 5b shows the selected IR images at *θ* = 0°, 45°, 90°, 135°, which are essential to calculate the Stokes parameters related to the linear polarization (S_0_, S_1_, S_2_) and the degree of linear polarization (DoLP). The IR images (for the single- and the double-layer Au gratings) collected at *θ* = 0° are much brighter than those collected at *θ* = 45°, 90°, 135° because unpolarized IR light (emitted by a blackbody) transmitted through the wire grid polarizer is linearly polarized perpendicular to the direction of Au grating. We can observe the drastic brightness-change of IR images at *θ* = 0°, 45°, 135°, depending on the sample structure (single- or double-layer Au grating structure). It can be estimated by the integral value of the transmission spectrum of the two structures (Fig. 2a) with respect to the wavelength (Details will be provided in the following section). Contrarily, both structures at *θ* = 90° show the darkest IR images since the transmitted light through the wire grid polarizer is linearly polarized parallel to the direction of Au grating (Fig. 2b). Note that it is difficult to distinguish the difference between the IR images of the two grating structures at *θ* = 90°. As will be addressed later, we extracted the digital output (voltage) from the pixels of our fabricated 320 × 256 MWIR type-II InAs/GaSb superlattice based focal plane array (FPA) device in order to accurately quantify the IR images at various mutual angles (*θ*). Also, the brightness in the IR images at *θ* = 45°, 135° can be expected to be $\sim 1/2$ of one at *θ* = 0° due to Malus’ law stating that the transmitted intensity (by the wire grid polarizer and the fabricated single- or double-layer Au grating) is proportional to the square cosine of the mutual angle ($\sim co{s}^{2}\theta$).

**Figure 6:**
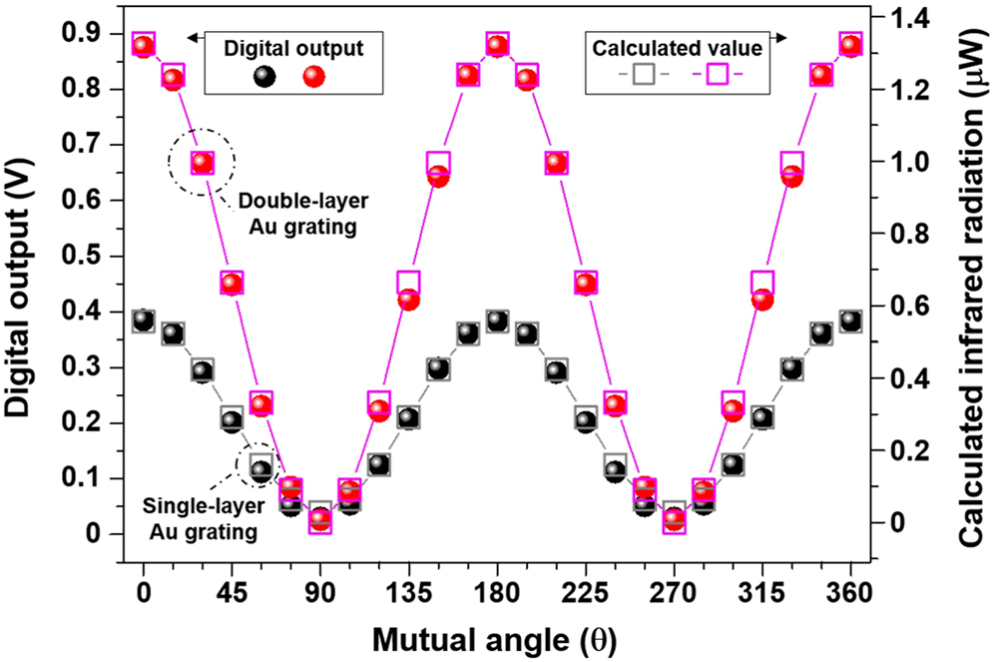
Digital output (voltage) extracted from the pixels of our fabricated 320 × 256 MWIR type-II InAs/GaSb superlattice based imager and calculated incidence power of the FPA device as a function of the mutual angle *θ* between two polarization axes of the fabricated Au grating and the wire grid polarizer.

Averaged output voltages over 100 pixels and calculated IR radiant flux onto the FPA device by varying the mutual angle $({0}^{\circ }\le \theta \,\le {360}^{\circ },\,\,{\rm{\Delta }}\theta ={15}^{\circ })$ are shown in Fig. 6. Note that our fabricated type-II superlattice based MWIR camera records a 320 × 256-pixel image, however the region that captured the metal target is composed of 10 × 10 pixels. The output voltages in Fig. 6 were adjusted by subtracting the background noise due to environmental temperature fluctuations from the measured ones^29^. The output voltages for the double-layer (single-layer) Au grating structure quantitatively agree well with Malus’ $co{s}^{2}\theta$ intensity law and are found to be 0.876 V (0.385 V) at *θ* = 0°, 180°, 360°; 0.448 V (0.202 V) at $\theta ={45}^{\circ }$; 0.422 V (0.208 V) at $\theta ={135}^{\circ }$; 0.026 V (0.029 V) at $\theta ={90}^{\circ },\,{270}^{\circ }$. Additionally, the degree of linear polarization (DoLP) can be calculated by $\sqrt{{S}_{1}^{2}+{S}_{2}^{2}}/{S}_{0}$, where ${S}_{0}={I}_{{0}^{\circ }}+{I}_{{90}^{\circ }},\,{S}_{1}={I}_{{0}^{\circ }}-{I}_{{90}^{\circ }},\,{S}_{2}={I}_{{45}^{\circ }}-{I}_{{135}^{\circ }}$ (*I*_*θ*_ is the intensity of polarized light at *θ*, so it can be considered as the averaged output voltage *V*_*θ*_), in case that the circularly polarized components can be ignored (i.e., *S*_3_ =0)^3,5^. The DoLP of the double (single)-layer Au grating structure is ~0.94 (0.86) with *S*_0_ = ~0.90 (0.41), *S*_1_ = ~0.85 (0.36), *S*_2_ = ~0.027 (−0.006). We find that the extinction ratio $({V}_{\theta ={0}^{\circ }}/{V}_{\theta ={90}^{\circ }})$ using the captured IR images (i.e., measured output voltages shown in Fig. 6) is obtained with ~14 and ~86 for the single- and the double-layer Au grating structures, respectively. The captured IR images (Fig. 5b) and extracted output voltages (Fig. 6) confirm that the double-layer Au grating structure provides a superior ability to detect the target with linear polarization states, which can be achieved by the enhanced TM transmission (*θ* = 0°) due to FP cavity resonance and by the highly reduced TE transmission (*θ* = 90°) due to the stacking of the subwavelength gratings.

Moreover, the IR radiant flux (*P*_*in*_) incident onto the camera was calculated using the following equation to anticipate the measured output and further details on the calculated data are presented in Fig. S3 (Supplementary Information).

3 $${P}_{in}=\,\frac{{A}_{BB}{A}_{grating}}{\pi {R}^{2}}{\int }_{{\lambda }_{1}}^{{\lambda }_{2}}M(\lambda ,\,T)\cdot {T}_{WGP}(\lambda )\cdot {T}_{grating}(\lambda )d\lambda $$

where *A*_*BB*_ and *A*_*grating*_ are the aperture area of the blackbody and the sample area, *R* is the distance between the blackbody and the MWIR camera, *T*_*WGP*_ is the transmission of the wire grid polarizer, and *T*_*grating*_ is the simulated transmission of double (single)-layer Au grating (with the geometrical dimensions of fabricated samples). The spectral radiant exitance of the blackbody, $M(\lambda ,T)$ is given by $M\,(\lambda ,T)={C}_{1}/{\lambda }^{5}(\exp ({C}_{2}/\lambda T)-1)$, where ${C}_{1}=3.74697\times {10}^{-16}W\cdot {m}^{2}$, ${C}_{2}=1.43941\times {10}^{-2}mK$, and $T$ is the blackbody temperature of 300 °C. $M\cdot {T}_{WGP}\cdot {T}_{grating}$ was integrated over the MWIR range, i.e., *λ*_1_ = 3 μm and *λ*_2_ = 5 μm in equation (3). The overall agreement between the experiment (measured output voltage) and the calculated IR radiant flux (*P*_*in*_) is apparent from Fig. 6.

## Conclusion

We have nanoimprint-lithographically fabricated a highly polarization-sensitive, dielectric cavity bounded between two layers of gold grating. The architecture of the double-layer Au grating brings the advantages of stacking 1D subwavelength gratings, such as the sequential attenuation of the linearly polarized light parallel to the grating direction (TE or $\theta ={90}^{\circ }$), while maintaining the advantages of an optical cavity exhibiting FP resonance modes, such as the enhanced transmission of linearly polarized light perpendicular to the grating direction (TM or $\theta ={0}^{\circ }$). The ratio of TM- to TE-transmitted light intensity for the double-layer grating structure is experimentally × 8 higher in the MWIR region as compared to the single-layer Au grating. This FTIR-measurement result is supported by the multiple-layer model based on a transfer matrix method. To demonstrate the capability to sense a linearly-polarized target, the radiometric characterization of the double-layer Au grating sample has been undertaken using a blackbody source along with a commercial wire-grid polarizer and a metal target. As a result, the ratio of ${V}_{\theta ={0}^{\circ }}$ to ${V}_{\theta ={90}^{\circ }}$ is significantly enhanced by more than 600%. Our findings suggest that the double-layer Au grating structure is very promising as a superior microgrid polarizer for the next generation high-performance polarimetric imaging systems.

## Electronic supplementary material

Supplementary information
