# Supplementary material for: Fabry-Perot cavity resonance enabling highly polarization-sensitive double-layer gold grating
Source: Sci Rep. 2018 Oct 3;8:14787. doi: 10.1038/s41598-018-32158-y (PMC6170388; doi:10.1038/s41598-018-32158-y)
Supplement: Supplementary file 1 — Supplementary information [file 41598_2018_32158_MOESM1_ESM.docx]

Supporting Information:

Fabry-Perot cavity resonance enabling highly polarization-sensitive double-layer gold grating

Jehwan Hwang^1,2,†^, Boram Oh^1,3,†^, Yeongho Kim^1,†^, Sinhara Silva^4^, Jun Oh Kim^1^, David A. Czaplewski^5^,

Jong Eun Ryu^3,6^, Eun Kyu Kim^2^, Augustine Urbas^7^, Jiangfeng Zhou^4,*^, Zahyun Ku^7,*^, and Sang Jun Lee^1,*^

**Fabrication of Si master mold and PDMS imprinting mold**


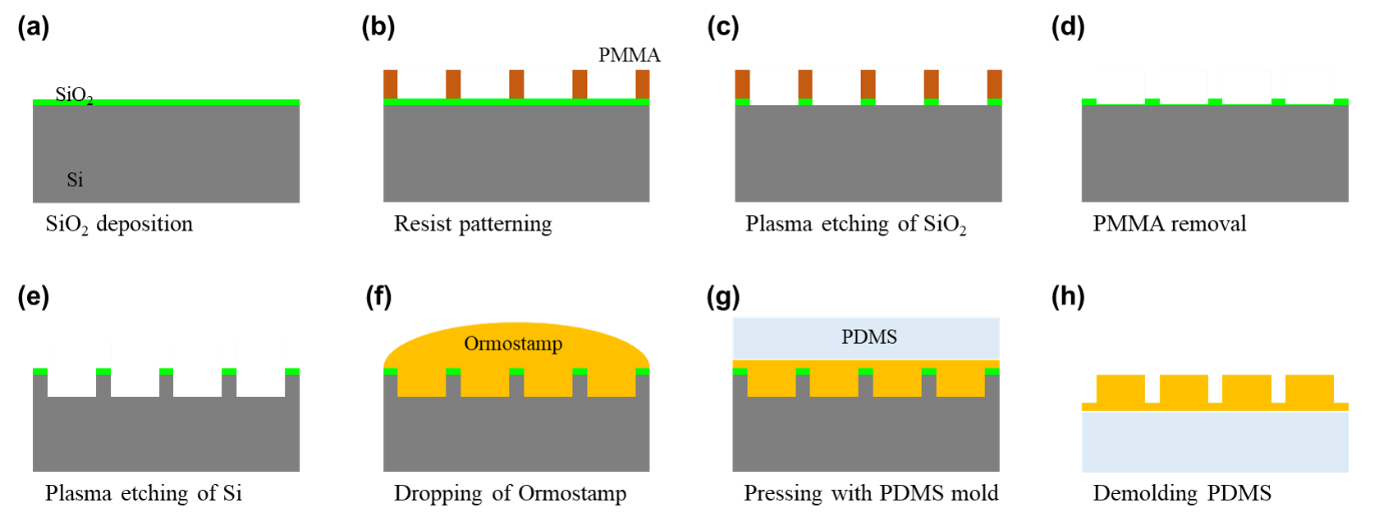


**Figure S1.** Schematic diagram of the fabrication of Si master mold and PDMS imprinting mold. (**a**) SiO_2_ deposition on Si substrate, (**b**) resist patterning by EBL process, (**c**) RIE of SiO_2_ using CF_4_ plasma, (**d**) PMMA resist removal in acetone, (**e**) RIE of Si using CF_4_/O_2_ plasma, (**f**) dropping of Ormostamp on Si master mold, (**g**) UV curing of Ormostamp, (**h**) demolding PDMS mold from Si master mold.

Figure S1 shows the fabrication process of a silicon (Si) master mold and an imprinting mold for ultraviolet-nanoimprint lithography (UV-NIL). A 80 nm-thick SiO_2_ was first deposited on a Si substrate using plasma enhanced chemical vapour deposition (PECVD). A 300 nm-thick polymethylmethacrylate (PMMA) electron beam resist (ER) was spun at 5000 rpm and soft-baked on a hotplate at 150°C for 1 min to obtain a resist thickness of ~300 nm. The resist grating patterns with periodicity (*p* = 1.0 μm) and width (*w* = 0.7 μm) were defined by electron-beam lithography (EBL) process. The exposed SiO_2_ was etched by a reactive ion etch (RIE) using an CF_4_ plasma and then the patterned resist was removed in acetone, followed by a RIE of Si with an CF_4_/O_2_ plasma. The fabricated Si master mold was coated with tridecafluoro-(1,1,2,2)-tetrahydrooctyl-trichlorosilane (F13-TCS) to form a hydrophobic surface. Ormostamp was spin-coated on the Si master mold and was brought into contact with a polydimethylsiloxane (PDMS) mold. After curing the Ormostamp by UV flood exposure, the PDMS imprinting mold was successfully demolded from the Si mater mold.

**Fabrication of double-layer Au grating**


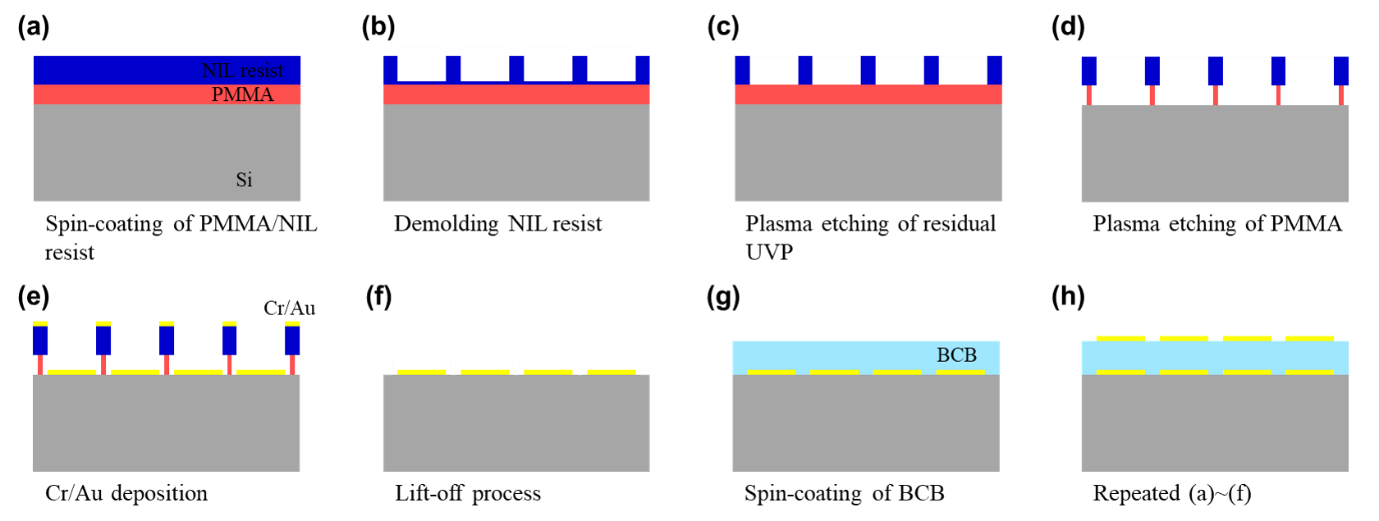


**Figure S2.** Schematic diagram of the fabrication of double-layer Au grating. (**a**) spin-coating of PMMA/UV-curable NIL resist on Si substrate, (**b**) nanoimprinting of grating patterns onto NIL resist and UV resist curing, (**c**) RIE of residual NIL resist using CHF_3_/O_2_ plasma, (**d**) RIE of PMMA resist using O_2_ plasma, (**e**) Cr (5 nm)/Au (100 nm) metallization, (**f**) lift-off process of PMMA/NIL resist in acetone, (**g**) spin-coating of BCB on lower Au grating/Si substrate, (**h**) the vertically aligned upper Au grating.

The double-layer Au grating was fabricated on a Si substrate using UV-NIL process as shown in Fig. S2. A 300 nm-thick PMMA resist was spin-coated on a silicon substrate and soft-baked on a hotplate at 180°C for 90 sec. A UV-curable NIL resist (UVP, EZimprinting) was then spin-coated onto the PMMA. The grating patterns on the PDMS mold were imprinted to the NIL resist at a pressure of 10 psi for 2 min by using a nanoimprinter (PL-400, EZimprinting) and the resist was UV-cured. The residue of the UV-cured resist and the PMMA resist were etched by a reactive ion etch (RIE) using CHF_3_/O_2_ and O_2_ plasma, respectively. Electron beam evaporation of Cr (5 nm)/Au (100 nm) and lift-off process of the PMMA/NIL resist were conducted to fabricate the lower Au grating. Benzocyclobutene (BCB) was diluted with T1100 rinse solvent at a volume ratio of 5:6 (BCB: T1100), spin-coated, and cured on a hotplate at 250°C for 1 hour to obtain a BCB thickness of 0.25 μm. Finally, the vertically aligned upper Au grating was fabricated on the BCB using the same NIL process condition.

**Linear polarization performance characterized by MWIR imager**


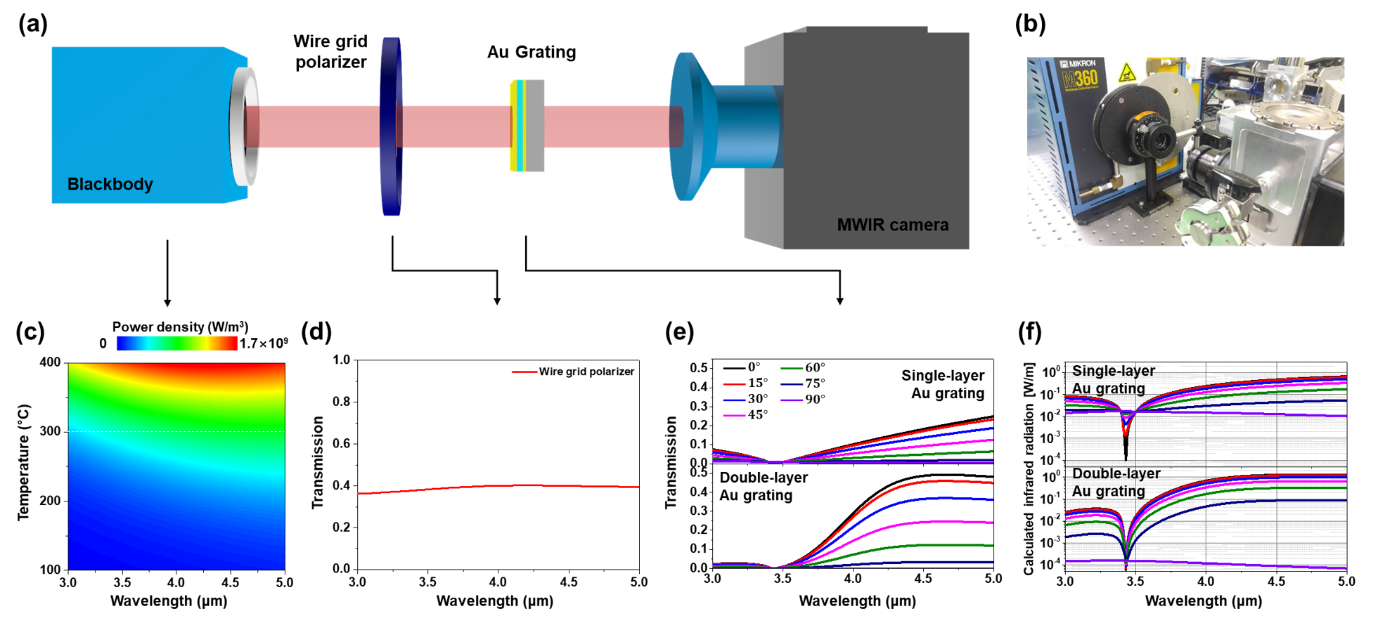


**Figure S3.** (**a**) Illustration of the experimental set-up, (**b**) picture of the set-up for the polarimetric imaging system with a blackbody, a wire grid polarizer, a metal target, a fabricated Au grating, and a T2SL based MWIR camera, (**c**) blackbody radiation as a function of wavelength and temperature, (**d**) measured transmission of the wire grid polarizer, (**e**) simulated transmission of the single-/double-layer Au grating, (**f**) calculated IR radiation incident on the MWIR camera.

The experimental set-up for characterization of the linear polarization performance of single-/double-layer Au grating structures is shown in Fig. S3a,b. Mid-wavelength infrared (MWIR) type-II InAs/GaSb superlattice (T2SL) photodetectors were grown on a GaSb substrate using a solid-source molecular beam epitaxy (MBE) system. The MWIR T2SL focal plane array (FPA) with 320×256 detector elements and 30 μm pitch was hybridized to an ISC9705 read-out integrated circuit (ROIC) chip (FLIR Systems, Goleta, CA) by flip-chip bonding and substrate removal. The blackbody temperature was selected as 300°C by taking into account the distance from the blackbody to the MWIR camera and the transmissions (*T_WGP_*(*λ*) and *T_grating_*(*λ*)) of the wire grid polarizer and the Au gratings) in the optical pathway. The IR radiation (*M*(*λ*, *T*)) from the blackbody was linearly polarized after passing through the wire grid polarizer and was then irradiated onto the fabricated grating samples. A metal target was placed between the wire grid polarizer and the fabricated sample to acquire linear polarization information. The transmission of the fabricated grating samples was simulated according to the angle of polarization being varied from 0° to 90°, with a step of 15°. The IR radiation incident on the MWIR camera was calculated by integrating the multiplication of *M*, *T_WGP_*, and *T_grating_* over the MWIR range of 3–5 μm.
